# Combined – whole blood and skin fibroblasts- transcriptomic analysis in Psoriatic Arthritis reveals molecular signatures of activity, resistance and early response to treatment

**DOI:** 10.3389/fimmu.2022.964274

**Published:** 2022-09-08

**Authors:** Alexandros Grivas, Maria Grigoriou, Nikos Malissovas, George Sentis, Anastasia Filia, Sofia Flouda, Pelagia Katsimpri, Panayotis Verginis, Dimitrios T. Boumpas

**Affiliations:** ^1^ Laboratory of Autoimmunity and Inflammation, Center of Clinical, Experimental Surgery and Translational Research, Biomedical Research Foundation Academy of Athens, Athens, Greece; ^2^ 4th Department of Internal Medicine, Attikon University Hospital, National and Kapodistrian University of Athens Medical School, Athens, Greece; ^3^ Immunohematology Laboratory, Democritus University of Thrace (DUTH), Alexandroupolis, Greece; ^4^ Institute of Molecular Biology and Biotechnology, Foundation for Research and Technology, Heraklion, Greece; ^5^ Laboratory of Immune Regulation and Tolerance, Division of Basic Sciences, University of Crete Medical School, Heraklion, Greece

**Keywords:** Psoriatic arthritis, transcriptome, molecular signatures, skin fibroblast, response to treatment

## Abstract

**Background:**

An interplay between immune cells and resident skin and joint stromal cells is implicated in psoriatic arthritis (PsA), yet the mechanisms remain elusive with a paucity of molecular biomarkers for activity and response. Combined transcriptomic and immunophenotypic analysis of whole blood and skin fibroblasts could provide further insights.

**Methods:**

Whole blood RNA-seq was performed longitudinally in 30 subjects with PsA at the beginning, one and six months after treatment, with response defined at six months. As control groups, 10 healthy individuals and 10 subjects with rheumatoid arthritis (RA) were recruited combined with public datasets from patients with psoriasis (PsO) and systemic lupus erythematous (SLE). Differential expression analysis and weighted gene co-expression network analysis were performed to identify gene expression signatures, while deconvolution and flow cytometry to characterize the peripheral blood immune cell profile. In a subset of affected and healthy individuals, RNA-seq of skin fibroblasts was performed and subjected to CellChat analysis to identify the blood-skin fibroblast interaction network.

**Results:**

PsA demonstrated a distinct “activity” gene signature in the peripheral blood dominated by TNF- and IFN-driven inflammation, deregulated cholesterol and fatty acid metabolism and expansion of pro-inflammatory non-classical monocytes. Comparison with the blood transcriptome of RA, PsO, and SLE revealed a “*PsA-specific signature*” enriched in extracellular matrix remodeling. This was further supported by the skin fibroblast gene expression profile, displaying an activated, proliferating phenotype, and by skin-blood interactome analysis revealing interactions with circulating immune cells through WNT, PDGF and immune-related semaphorins. Of note, resistance to treatment was associated with upregulation of genes involved in TGFβ signaling and angiogenesis and persistent increase of non-classical monocytes. Differentially expressed genes related to platelet activation and hippo signaling discriminated responders and non-responders as early as one month after treatment initiation.

**Conclusion:**

Transcriptome analysis of peripheral blood and skin fibroblasts in PsA reveals a distinct disease activity signature and supports the involvement of skin fibroblasts through their activation and interaction with circulating immune cells. Aberrant TGFβ signaling and persistently increased non-classical monocytes characterize treatment-resistant PsA, with pro-inflammatory pathways related to platelet activation and Hippo signaling predicting early response to treatment.

## Introduction

Psoriatic arthritis (PsA) is a chronic, musculoskeletal disease that develops in up to one-third of patients with cutaneous psoriasis (PsO) (1, 2). PsA manifests with inflammation of the peripheral joints, the entheses and the spine, and is uniquely characterized by synchronous bone erosions and new bone formation (3). PsA patients also display a wide spectrum of extra-articular features such as uveitis and colitis, and an increased risk of cardiometabolic comorbidities, including obesity and hyperlipidemia (4). These comorbid conditions increase the overall disease burden leading to poor function and quality of life (5).

The advent of biological disease-modifying anti-rheumatic drugs (bDMARDs) targeting T cells, TNFα and IL-17/-23 axis has revolutionized PsA management, highlighting the role of these cells and cytokines in disease propagation (6). Despite this progress, approximately 40% of patients fail to optimally respond to these treatments (7, 8). This lack of efficacy highlights the molecular heterogeneity among PsA patients and the significant gap in our knowledge regarding mechanisms of treatment resistance in PsA. The myeloid cell compartment has been relatively understudied in PsA, although emerging evidence suggests an important role for these cells in driving joint and skin inflammation (9). Likewise, stromal cells, such as skin and synovial fibroblasts, have not yet been sufficiently characterized in PsA, despite standing at the forefront of research in other inflammatory arthritides (10, 11). Elucidating the role of these cells in disease pathophysiology along with identifying biomarkers of diagnostic and prognostic potential represent significant unmet needs in PsA.

Gene expression studies have provided significant insights into the complex pathogenetic mechanisms of autoimmune diseases and have paved the way towards precision medicine in the field of rheumatology (12). Transcriptomic studies in PsA have revealed signatures related to TNFα and IL-17 axis in peripheral blood and target tissues. Nevertheless, these studies have been performed using microarray technology and are limited in number, thus leaving the transcriptomic profile of PsA still underexplored (13).

In this study, we utilize high-throughput mRNA sequencing technology coupled with immunophenotyping to investigate the molecular landscape of blood cells and skin fibroblasts in PsA. We first identify an *activity signature* in blood characterized by TNF- and Interferon-mediated inflammation, lipid-related metabolic aberrancies, and expansion of non-classical monocytes (NCM). We also define a “*PsA-specific gene set*” related to extracellular matrix (ECM) metabolism, which is distinct in PsA compared to other autoimmune rheumatic diseases, such as Rheumatoid Arthritis (RA), Psoriasis (PsO), and Systemic Lupus Erythematosus (SLE). We perform longitudinal analysis in a subset of PsA patients delineating signatures of *resistance to treatment* associated with TGFβ signaling and angiogenesis as well signatures of *early response to treatment* pertaining to platelet activation and Hippo signaling. Finally, combined blood and skin fibroblasts’ network analysis identifies increased interactions between blood immune cells and skin fibroblasts, suggesting a novel role for these cells in disease pathophysiology. These findings have implications regarding our understanding of PsA pathogenesis as well as suggesting molecular biomarkers of diagnostic and prognostic potential.

## Materials and methods

### Experimental design and study participants

**Figure 1:**
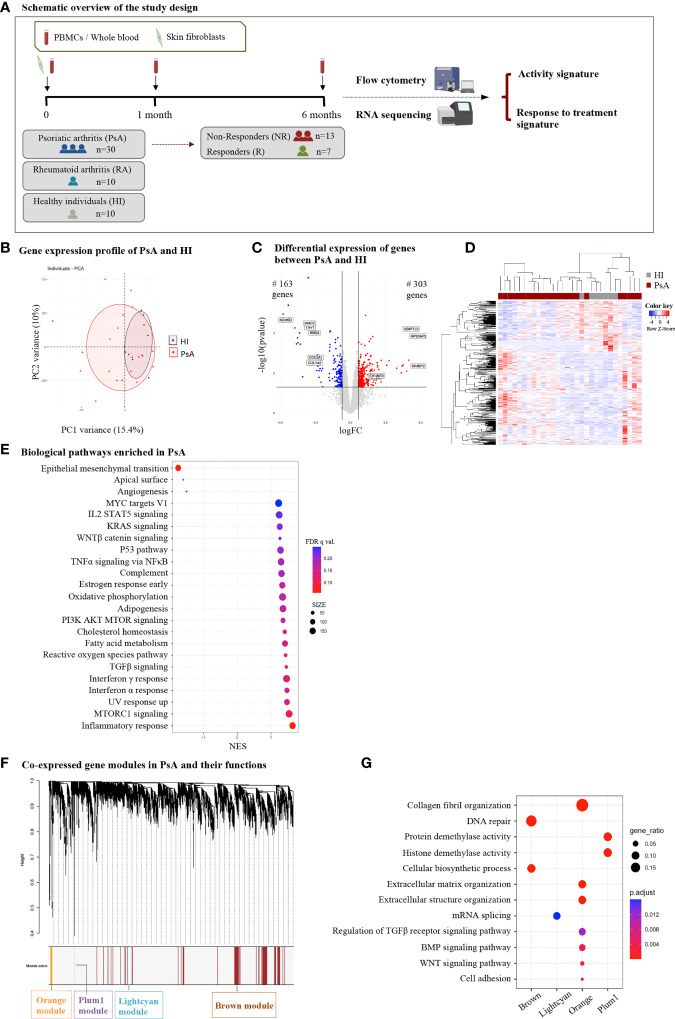
Subjects with PsA display widespread transcriptome perturbations in peripheral blood compared to healthy individuals. **(A) **Schematic representation of the study design. Subjects with active PsA were followed up for 6 months after treatment initiation being classified as R or NR. Blood samples were collected at baseline (0-), 1- and 6-month time points. A subset of three PsA patients donated skin biopsy samples for fibroblast isolation. RNA from whole blood and skin fibroblasts was analyzed with RNA sequencing to determine signatures of activity and response to treatment. Flow cytometry analysis of PBMCs was performed in parallel. RA patients and HI were also included in the study. **(B)** PCA of blood gene expression profiles from PsA patients (n=23) and HI (n=7). The two first principal components (PC1, PC2) are plotted. **(C)** Volcano plot and **(D)** heatmap of DEGs between PsA and HI. The up- and down-regulated genes are denoted by red and blue points, respectively. Gray points indicate genes with no significant difference. **(E)** Dot plot of GSEA analysis representing biological pathways associated with the Hallmark v7.5 database. The figure shows the positively and negatively enriched pathways in PsA. The size of the dots represents the number of genes included in each enriched term. **(F)** WGCNA analysis of blood gene expression data: Gene dendrogram obtained by average linkage hierarchical clustering and **(G)** dot plot demonstrating functional annotation of the gene modules. Gene ratio represents the ratio of gene count to term size. PsA, Psoriatic arthritis; R, Responders; NR, Non-responders; PBMCs, Peripheral blood mononuclear cells; RA, Rheumatoid arthritis; HI, healthy individuals; PCA, Principal component analysis; DEGs, differentially expressed genes; GSEA, gene set enrichment analysis; WGCNA, weighted gene co-expression network analysis; FC, fold change; NES, normalized enrichment score.

This is a prospective, longitudinal study of subjects with PsA recruited through the Rheumatology and Clinical Immunology Department of the Attikon University Hospital in Athens, Greece. Subjects with PsA were diagnosed according to the Classification Criteria for Psoriatic Arthritis (CASPAR). They displayed the polyarthritic phenotype of the disease and presented with moderate to high disease activity based on the DAPSA score (DAPSA >15) (14, 15). Subjects either started or switched treatment at baseline and were followed-up for a period of 6 months, when response to treatment was determined according to the ACR50 response and/or “75% change of DAPSA” (16). Blood samples were collected at baseline, 1-month, and 6-month time points and were used for peripheral blood mononuclear cell (PBMC) isolation and whole blood RNA isolation. Skin biopsies were obtained from the lesional skin of three PsA patients for fibroblast isolation. Blood from Healthy Individuals (HI, n=10) and subjects with Rheumatoid Arthritis (RA, n=10) was also collected. Individuals with RA were diagnosed according to the 1987 American College of Rheumatology (ACR) criteria and had severe disease activity (DAS28 > 5.1) according to the disease activity score based on the 28 joint counts. Written informed consent was obtained from all study participants and protocols for the procedures were approved by the hospital’s research ethic committee. All studies were conducted in accordance with ethical guidelines of the Declaration of Helsinki. The demographic and clinical characteristics of all participants are summarized in **Table 1**. An overview of the study workflow is outlined in **Figure 1A**.

**Table 1:** Demographic and clinical characteristics of healthy individuals, PsA and RA patients.

Characteristics*	HI(n=10)	PsA(n=30)	RA(n=10)	P value
**Female (%)**	7 (70%)	22 (73%)	8 (80%)	
**Age, years**	41.7 (13)	51.1 (10)	54.2 (10)	0.047
**Duration of arthritis, years**	-	6.2 (5.8)	4 (3.5)	0.58
**Duration of psoriasis, years**	–	20.3 (12.1)		
**Disease activity, indices**	-	DAPSA 46 (15.3)	DAS28 5.8 (0.4)	
**Enthesitis, n (%)**	–	11 (36%)	–	
**Dactylitis, n (%)**	-	3 (10%)	-	
**ESR, mm/hr**	–	23.1 (18.5)	33.6 (13.4)	0.2
**Current treatment, n**				
** Naïve**	-	11	0	
** DMARDs** (methotrexate, leflunomide, apremilast)		5	3	
** Biologic** (TNFi, secukinumab, ustekinumab)		14	4	
** Immuno-suppressants** (rituximab)			2	

*All values are presented as ‘mean (SD)’ unless otherwise stated. All variables had <20% missing data. HI, Healthy Individuals; PsA, Psoriatic Arthritis; RA, Rheumatoid Arthritis; DAPSA, Disease Activity Score for Psoriatic Arthritis; DAS28, Disease Activity Score in 28 joints; ESR, Erythrocyte Sedimentation Rate; DMARD, Disease Modifying Anti-rheumatic Drug; TNFi, Tumor Necrosis Factor inhibitors.

### Cell isolation

*Peripheral blood mononuclear cells (PBMCs)* were isolated by Ficoll (Lymphosep #L0560, Biowest) density gradient centrifugation and were cryopreserved in freezing medium.

*Skin fibroblasts* were isolated from samples of lesional skin from three subjects with PsA, obtained with a 4mm-punch biopsy. The subjects were two men and one woman who had active plaque psoriasis, diagnosed clinically by an expert dermatologist. Psoriasis severity was assessed using the PASI (Psoriasis Area and Severity Index) score (mean 2.4) and BSA (Body Surface Area) (mean 5%). Patients had received no topical treatment for two weeks before the procedure. Two subjects were on biologic treatment (anti-IL17, anti-IL-12/23 antibody, respectively) and one was treatment-naive at time of procedure. Skin biopsies were collected also from three age- and sex- matched healthy individuals. Following excision, skin tissue was washed in sterile PBS and sliced in 4-5 pieces. Tissue fragments were incubated at 37°C for two hours in a 15-ml falcon containing enzymes for digestion (DNAse 0.25mg/ml, dispase 40μg/ml, collagenase 1mg/ml). After incubation, the mixture was pipetted through a 70μm mesh cell strainer, cultured *in vitro* with DMEM (Dulbecco’s Modified Eagle Medium, Thermo Fischer Scientific), supplemented with 10% fetal bovine serum (Thermo Fischer Scientific) and penicillin/streptomycin (50 µg/mL, Thermo Fischer Scientific), in a humidified atmosphere of 5% CO_2_ at 37°C. Proliferating fibroblasts between passages 4-5 were used for the transcriptomic analysis.

### Flow cytometry

Flow cytometry was performed using a BD FACS-Aria-III (Becton Dickinson Biosciences) and analyzed using FlowJo v10 Software (BD Life Sciences, RRID : SCR_008520). To assess the frequency of cells of interest among the PBMCs, specimens were stained with the following conjugated antibodies (Biolegend) (clone, catalogue number): HLA-DR (L243, #307618, RRID : AB_493586), CD14 (M5E2, #301804, RRID : AB_314186), CD16 (3G8, #302012, RRID : AB_314212), CD33 (WM53, #303404, RRID : AB_314348), CD15 (W6D3, #323018, RRID : AB_893256), CD123 (6H6, #306017, RRID : AB_10900244), CD4 (OKT4, #317428, RRID : AB_1186122), CD8 (SK1, #344714, RRID : AB_2044006), CD127 (A019D5, #351316, RRID : AB_10900804), CD25 (BC96, #302604, RRID : AB_314274).

### RNA isolation and RNA sequencing pipeline

Total RNA from blood was extracted using the Tempus Spin RNA Isolation Reagent Kit (#4378926, Thermo Fischer), while RNA isolation from skin fibroblasts was performed using the NucleoSpin RNA Isolation Kit (#740955.250, Macherey-Nagel). RNA was purified as per the manufacturer’s protocol. For library preparation, we performed mRNA selection using NEBNext^®^ Poly(A) mRNA Magnetic Isolation Module (New England Biolabs) and subsequently prepared the libraries using NEBNext^®^ Ultra II Directional Library Preparation kit (New England Biolabs). Sequencing was performed on Illumina NextSeq 500 with single-end 75-bp reads. Quality of sequencing was assessed using FastQC (RRID : SCR_014583) (17). Raw fastq sequencing reads were aligned against the human reference genome sequencing (version hg38) using the STAR 2.6 algorithm (RRID : SCR_004463) (18). Gene quantification was performed using HTSeq 0.11 (RRID : SCR_005514) with -s reverse option and gencode v29 annotation file (19). Raw counts were corrected for sequencing batch effect using ComBat-seq in R (20). Differential expression (DE) analysis was performed using the edgeR software (qlmQLFtest function, RRID : SCR_012802) in R (21). Genes with fold change |FC|≥1.5 and P value<0.05 were selected as significantly DE genes (DEGs). Heatmaps and volcano plots were created with R using in-house developed scripts based on ggplot2 package (RRID : SCR_014601). Venn diagrams were created using Venny 2.1.0 (RRID: SCR_016561) (22)

### Enrichment analysis

To explore the function of DEGs, we performed pathway and gene ontology (GO) enrichment analysis using the g:Profiler web-server (RRID : SCR_006809) (23), identifying enriched pathways among the significant DEGs. Enriched pathways with Benjamini-Hochberg corrected P value ≤ 0.05 were considered statistically significant. Gene Set Enrichment Analysis (GSEA, RRID : SCR_003199) was also performed to reveal enriched signatures in our dataset. As a reference gene set we used the Molecular Signatures Database (MSigDB v7.5) (24). All expressed genes were ranked by descending value of the product of –log10(P-value) and FC. Highly upregulated genes were at the top and highly downregulated genes were at the bottom of the ranked list. GSEA pre-ranked analysis was then performed using the default settings. Gene set enrichment was considered significant when False Discovery Rate (FDR) <25%.

### Deconvolution of blood gene expression data

We used the CIBERSORTx deconvolution algorithm (RRID : SCR_016955) (25) to determine the proportion of immune cell subsets in blood. This algorithm uses the normalized gene expression values and a signature matrix to calculate the cell type frequencies of a sample. BAM alignment files, as derived from STAR algorithm, were used as input in Cufflinks 2.2.2 (RRID : SCR_014597) (26) using Ensembl v94 annotation file, and Fragments Per Kilobase Million (FPKM) expression values were generated. FPKM expression data was used as input to the CIBERSORTx web portal, along with the LM22 signature matrix to identify 22 infiltrating immune cell components, including: subsets of macrophages (M_0_, M_1_, and M_2_), T cells (CD8^+^, naïve CD4^+^, memory resting CD4^+^, memory activated CD4^+^, T_fh_ cells, regulatory T cells, and gamma delta T cells), natural killer (NK) cells (resting and activated NK cells), mast cells (resting and activated mast cells), B cells (naïve and memory B cells), dendritic cells (resting and activated dendritic cells), monocytes, plasma cells, neutrophils, and eosinophils. Results with CIBERSORTx Pvalue<0.05 were reserved for the following analysis.

### Construction of weighted gene co-expression network analysis

Weighted gene co-expression network analysis (WGCNA, RRID : SCR_003302) (27) was used to identify clusters of co-expressed genes in our blood gene expression dataset. The WGCNA package in R was utilized to construct a co-expression network based on the normalized expression data. A soft-threshold power of 7 was selected to achieve approximate scale-free topology (*R*2 ~ 0.79). Network was constructed using the blockwiseModules function. The function uses average linkage hierarchical clustering for the dendrogram construction and the Dynamic Hybrid tree-cutting method for module identification. Modules of co-expressed genes were labeled by color coding for illustration purposes, while genes that did not fall within a specific module were assigned the color gray. We performed functional annotation of the genes within each module using the g:Profiler web-server (23).

### Skin fibroblasts-whole blood interaction network

We utilized CellChat (RRID : SCR_021946) (28) to examine the crosstalk between blood cells and skin fibroblasts. CellChat is a tool developed for single cell RNA-seq datasets, capable of inferring communication networks by combining a curated ligand-receptor database with statistical tests, mass action models, and gene expression data. By employing our blood and skin fibroblasts’ gene expression profiles, CellChat software modeled the communication probability between these two compartments. Bulk RNA-seq data were used, and therefore only signals received or sent by fibroblasts were identified without knowing which specific blood cell type they interact with.

### Data analysis and statistics

Results are presented as mean ± sd. Data between two groups were compared using the two-tailed, Student’s t-test or the two-tailed, Mann–Whitney U-test, as appropriate (after testing for normality with the F test). Data between three groups were compared using the ordinary one-way ANOVA or the Kruskal-Wallis test, as appropriate. The specific statistical tests performed are indicated in the figure legends. All statistical analyses were performed on GraphPad Prism software (v9.0.0, RRID : SCR_002798). P values <0.05 were considered as statistically significant.

### Data availability statement

The RNA-seq data from blood and skin fibroblasts can be found in the EGA repository under the accession number EGAS00001006288.

## Results

### Transcriptomic profiling of PsA peripheral blood identifies perturbations related to inflammation, metabolism and collagen biosynthesis

To identify genes and molecular pathways involved in the pathogenesis of PsA, we first compared the blood gene expression profile of subjects with PsA (n=23) and healthy individuals (HI, n=7). Despite significant overlap in the PCA (**Figure 1B**), differential expression analysis identified 466 DEGs (|FC|>1.5, P value<0.05), of which 303 were up- and 163 were down-regulated in PsA as compared to HI (**Figure 1C, D**). To understand the biological differences between PsA and healthy state, GO and pathway enrichment analysis were performed. GSEA indicated positive enrichment in PsA for several biological pathways related to inflammation and the immune system, such as the *inflammatory response*, *TNFa signaling via NFκB*, *complement*, *IL2 signaling*, *IFNα* and *IFNγ response*. Metabolic pathways including *oxidative phosphorylation, adipogenesis, fatty acid metabolism* and signaling cascades related to *WNTβ catenin, TGFβ* and *MTORC1* were also positively enriched in PsA. On the other hand, negative enrichment was identified in pathways related to *epithelial mesenchymal transition* and *angiogenesis*. (**Figure 1E**).

To further disentangle the transcriptomic profile of PsA blood, we utilized Weighted Gene Co-expression Network Analysis (WGCNA). Through this approach, DEGs were classified into four statistically significant modules (**Figure 1F**). Among them, the *orange* module was most closely associated with PsA and included 110 genes related to e*xtracellular matrix (ECM) organization*, *cell adhesion*, *BMP-* and *WNT-mediated signaling*. The other three modules -*lightcyan* (251 genes), *brown* (1569 genes), and *plum1* (138 genes)- showed enrichment in processes related to *mRNA splicing, DNA repair*, *cellular biosynthesis*, and *demethylation* (**Figure 1G**). Collectively, these findings suggest that the blood transcriptome in PsA is characterized by TNF- and Interferon-driven inflammation, metabolic perturbations and aberrant ECM remodeling, indicating that these processes are critical to sustaining disease activity.

### Immune profiling of peripheral blood reveals expansion of non-classical monocytes during active disease

To better characterize the cellular landscape of active PsA, we performed phenotypic characterization of the blood immune cell compartment in patients and healthy donors. First, we used CIBERSORTx to analyze the immune cell fraction based on the blood gene expression profile (**Supplementary Figure 1A**). The result of the deconvolution analysis for 13 cell subsets indicated a higher proportion of monocytes and a lower proportion of neutrophils in PsA compared to healthy state. However, both differences did not reach the statistically significant threshold.

**Figure 2:**
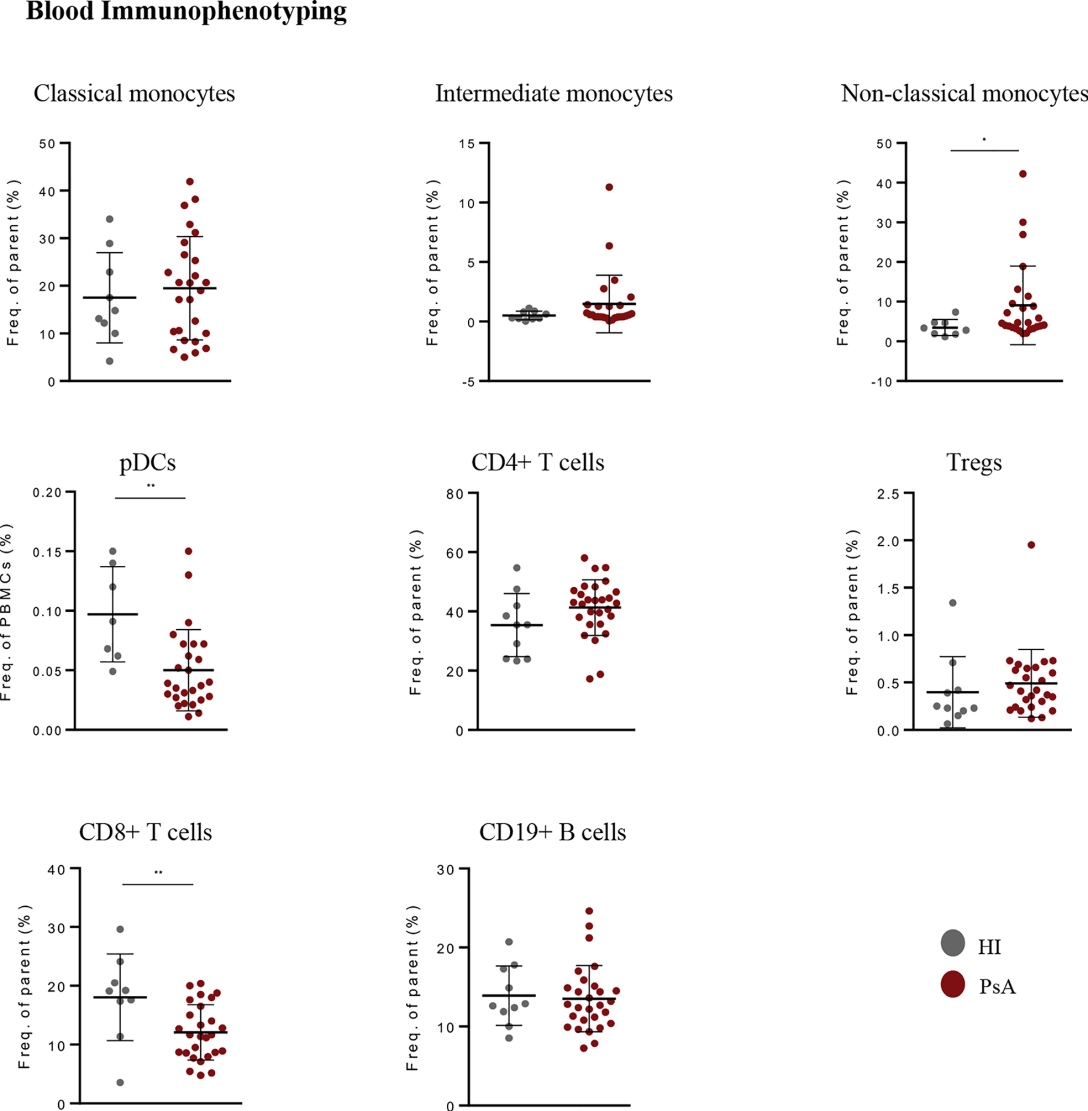
Immunophenotyping in blood of PsA patients and healthy individuals. Frequencies of monocyte subsets, pDCs, B cells, Tregs, CD4+ and CD8+ T cells in peripheral blood of PsA patients (n=26-30) and HI (n=8-10). Results are demonstrated as mean with SD. Statistical significance was obtained by unpaired Student’s t-test and Mann-Whitney test. (*p ≤ 0.05, **p ≤ 0.01, ***p ≤ 0.001). PsA, Psoriatic arthritis; HI, healthy individuals; pDCs, plasmacytoid dendritic cells; Tregs, T regulatory cells.

Next, we performed flow cytometry analysis to characterize specific myeloid and lymphoid cell subsets in the peripheral blood of PsA (n=30) and HI (n=10) (**Figure 2A**, **Supplementary Figure 1B**). An additional subset of 10 subjects with rheumatoid arthritis (RA) was recruited as a disease control group. We found increased frequency of the non-classical monocytes (NCM) in PsA compared to healthy subjects, in line with the results of the deconvolution analysis. Additionally, we observed lower frequencies of circulating pDCs and CD8^+^ T cells in PsA. Interestingly, PsA patients shared a similar blood immune profile with RA patients (**Supplementary Figure 1C**). Together, these data suggest expansion of the pro-inflammatory monocytic subset in PsA blood during active disease and reduction of blood pDCs and cytotoxic T cells, probably due to their selective migration at the sites of inflammation.

### PsA is characterized by a distinct blood signature denoting ECM metabolism and aberrant blood-skin fibroblasts cross-talk

**Figure 3:**
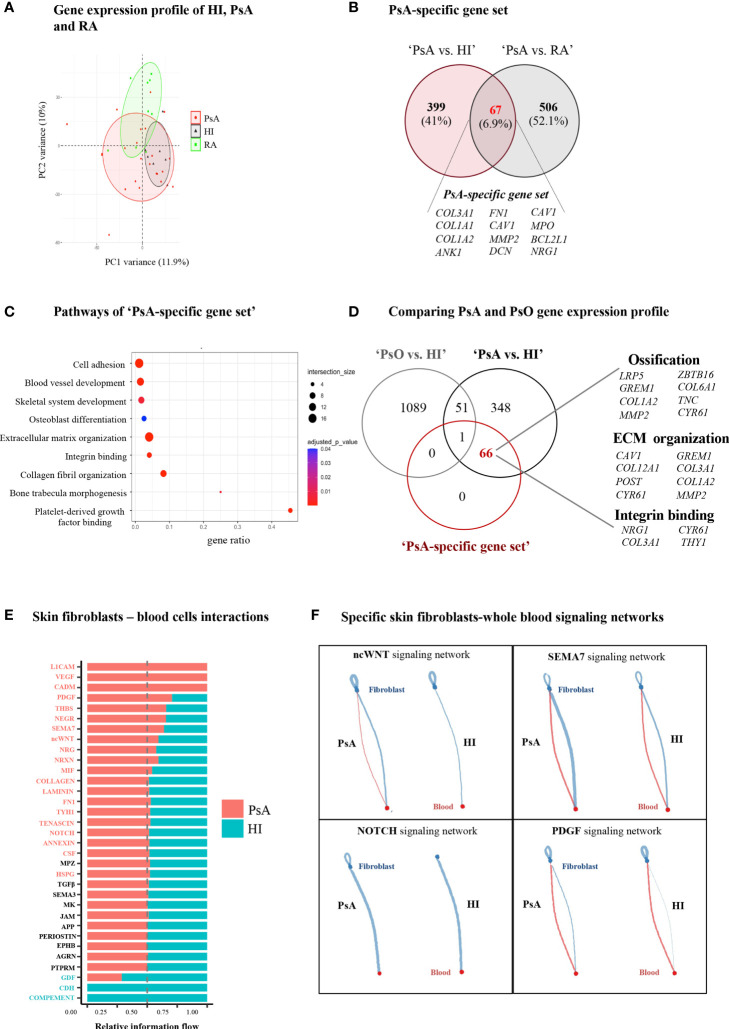
PsA displays a disease-specific signature in blood related to enhanced ECM turnover. **(A)** PCA of blood gene expression profiles from PsA, RA and HI. The two first principal components (PC1, PC2) are plotted. **(B)** Venn diagram showing the overlap between ‘PsA vs. HI’ and ‘PsA vs. RA’ DEGs, representing the “PsA-specific gene set”. Selected genes are listed at the bottom of the diagram. **(C)** Gene ontology analysis, based on g:Profiler, for functional annotation of the “PsA-specific gene set”. **(D)** Venn diagram showing the overlap among ‘PsA vs. HI’ DEGs, ‘PsO vs. HI’ DEGs and the ‘PsA-specific gene set’. Selected genes related to integrin binding, ECM organization and ossification are depicted. **(E)** Bar plot illustrating the interaction pathways between blood cells and skin fibroblasts in psoriatic and healthy samples. Significance is inferred based on the relative information flow in each interaction pathway. Red denotes signaling pathways enriched in PsA, blue denotes signaling pathways enriched in healthy state, while pathways depicted in black are equally enriched between the two states. **(F)** Plots showing selected signaling pathways between blood cells and skin fibroblasts in PsA and HI. Lines originate from a cell type (blood cells or fibroblasts), indicating the source of the ligand, and connect to the cell type (fibroblasts or blood cells, respectively) where the receptors are expressed. The width of each line is proportional to the communication probability, inferred by the number of unique ligand-receptor interactions. Loops represent autocrine circuits. ECM, Extracellular matrix; PsA, Psoriatic arthritis; HI, Healthy individuals; RA, Rheumatoid arthritis; DEGs, Differentially expressed genes; PsO, Psoriasis.

We next investigated whether a specific transcriptomic signature in blood could characterize PsA compared to other inflammatory arthritides and autoimmune diseases. To this end, we performed whole blood RNA-seq analysis in a group of patients with active RA (n=8), a disease that shares many clinical and molecular features with PsA (**Figure 3A**, **Supplementary Figure 2A, B**). The intersection of DEGs in ‘PsA vs. HI’ and ‘PsA vs. RA’ comparisons converged to a panel of 67 genes specific for PsA, comprising the “*PsA-specific gene set*” (**Figure 3B**, **Supplementary Table 1**). These genes were enriched in *cell adhesion*, *blood vessel development* and pathways related to ECM metabolism such as *ECM organization*, *collagen fibril organization* and *bone trabecula morphogenesis* (**Figure 3C**). Of note, most of these “PsA-specific gene set” were downregulated in the ‘PsA vs. HI’ comparison, probably implying the efflux of circulating mesenchymal-like cells from the blood to affected tissues during a PsA flare.

Given that PsA is the most common and severe complication of psoriasis (PsO), we next sought to investigate whether this “PsA-specific gene set” could also discriminate arthritis from cutaneous psoriasis. To address this, we utilized a publicly available gene expression dataset derived from the blood of subjects with well-characterized general pustular PsO (Catapano et al) (29). Comparing the ‘PsA-specific gene set’ with the ‘PsA vs. HI’ and ‘PsO vs. HI’ DEGs, we identified 66 out of the 67 “PsA-specific genes” remaining related only to PsA (**Figure 3D**). A similar analysis utilizing publicly available data from patients with Systemic Lupus Erythematosus (SLE) (30) resulted in 57 genes being uniquely represented in PsA (**Supplementary Figure 3**). Hence, these findings suggest that a blood signature related to ECM metabolism and remodeling is highly specific to PsA.

Following the identification of a specific ECM-related signature in PsA blood, we next examined the contribution of stromal cells –the primary ECM regulators- to disease activity signature. Skin is an easily accessible target tissue in PsA, and thus we focused our analysis on skin fibroblasts. Specifically, we performed RNA-seq in skin fibroblasts obtained from three subjects with PsA and three HI. PsA fibroblasts demonstrated a distinct gene expression profile compared to healthy fibroblasts (**Supplementary Figures 4A**), being enriched in *oxidative phosphorylation* and cell cycle-related processes such as *G2M checkpoint and E2F targets* (**Supplementary Figures 4B**). Integrating the blood and skin transcriptomes through CellChat we inferred, the signaling networks between the blood immune cells and skin fibroblasts. Interaction networks were increased in number and strength in PsA compared to healthy state and included signaling cascades regulated by immune related semaphorin (*SEMA7*), *ncWNT*, *PDGF* and *NOTCH* (**Figure 3E, F**, **Supplementary Figure 4C**). Altogether, these findings suggest that skin stromal cells display an activated, proliferating phenotype in PsA, supporting a network of interactions with circulating immune cells.

### Longitudinal immunophenotypic and transcriptomic analysis reveals distinct signatures associated with early response and resistance to treatment

**Figure 4:**
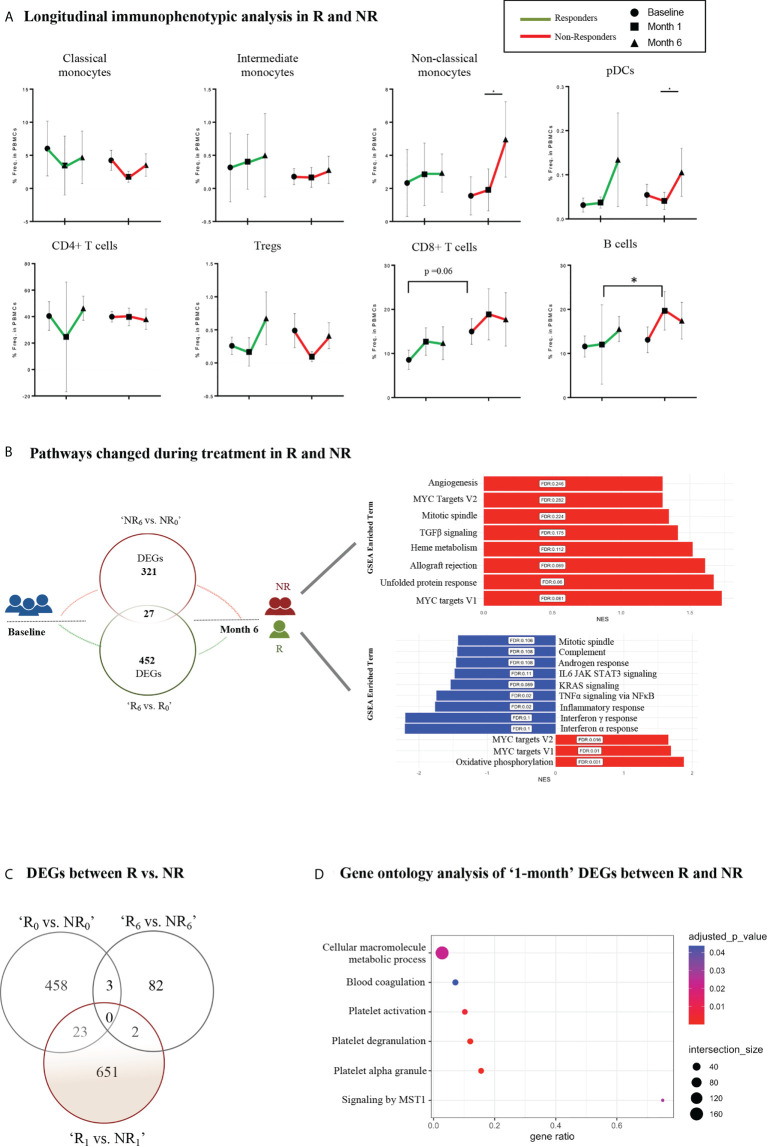
Longitudinal immunophenotypic and transcriptomic analysis in Responders and Non-Responders. **(A)** Frequencies of immune cell populations in R and NR (myeloid subsets–up, lymphoid subsets–down) at baseline (0-), 1- and 6-month time points. Results are demonstrated as mean with SD. Statistical significance was obtained by two-way ANOVA test. **(B)** Schematic diagram of the molecular changes over the course of the follow-up period. Venn diagram (left) represents the DEGs between baseline (0-) and 6-month in R and NR, respectively. The bar plots (right) show the corresponding changes in molecular pathways in NR (up) and R (down). **(C)** Venn diagram representing DEGs between R vs. NR at baseline (0-), 1- and 6-month after treatment initiation. **(D)** Dot plot representing pathway analysis of DEGs from R vs. NR at 1-month time point after treatment initiation based on g:Profiler database. R, Responders; NR, Non-Responders; DEGs, Differentially expressed genes. *p ≤ 0.05.

To explore molecular biomarkers of prognostic potential in PsA, we performed longitudinal assays in a subset of patients (n=20) and investigated differences between responders (R, n=7) and non-responders (NR, n=13) at different time points after initiation of treatment (**Supplementary Table 2**). Both patient groups showed similar clinical manifestations at baseline and 1-month, suggesting the absence of clinical predictors of response to treatment in our cohort (**Table 2**). Immunophenotypic analysis revealed increased frequency of non-classical monocytes in NR at the ‘6- month vs. 1-month’ time point, indicating a persistence of these cells despite treatment (**Figure 4A**).

**Table 2:** Baseline clinical characteristics of PsA patients according to ACR50/DAPSA75% response at 6 months after treatment.

Characteristics*	All patients(n=30)	Complete follow up (n=20)		P Value
		Responders(n=7)	Non- Responders (n=13)	
**Female (%)**	22 (73%)	5 (71%)	8 (61%)	
**Age, years**	51.1 (10)	49.4 (9.5)	51.6 (9)	0.6
**Duration of arthritis, years**	6.2 (5.8)	7.2 (7.8)	7 (1.2)	0.9
**Duration of psoriasis, years**	20.3 (12.1)	27 (10)	19.3 (10.5)	0.1
**Baseline disease activity by DAPSA**	46 (15.3)	45.7 (17.5)	46.8 (15.5)	0.8
**ESR (mm/hr)**	23.1 (18.5)	25.4 (19.6)	20 (15)	0.4
**Baseline treatment, n**				
** Naïve**	11	2	4
** DMARDs**	5	2	1
** Biologics**	14	3	8

*All values are presented as ‘mean (SD)’ unless otherwise stated. All variables had <20% missing data. PsA, Psoriatic Arthritis; DAPSA, Disease Activity Score for Psoriatic Arthritis; ESR, Erythrocyte Sedimentation Rate; DMARDS, Disease Modifying Anti-Rheumatic Drugs.

Longitudinal gene expression profiling revealed significant transcriptomic differences in the blood between R and NR (**Supplemental Figure 5A**). Specifically, after the initiation of treatment, responders demonstrated downregulation of pathways related to inflammation, such as *TNFα signaling via NFκB*, *KRAS signaling*, *complement*, *IFNα* and *IFNγ response* (**Figure 4B**, **Supplemental Figure 5B**). On the other hand, NR showed upregulation of genes associated with *angiogenesis*, *TGFβ signaling* and *mitotic spindle.* Notably, genes involved in MYC signaling were overexpressed in both patient groups (**Figure 4B**, **Supplemental Figure 5B**). Additionally, comparison of the gene expression profile of R and NR at the different time points revealed significant differences as early as 1-month after treatment implementation (**Figure 4C**). Specifically, 651 DEGs enriched in *cellular macromolecule metabolic process*, *MST1-mediated signaling*, *platelet activation* and *degranulation* comprised a gene signature that could differentiate the two groups according to their response early during their treatment course (**Figure 4C, D**, **Supplemental Figure 5C**). Collectively, these findings highlight the molecular heterogeneity of PsA and suggest a novel molecular stratification of patients based on their gene expression profile.

## Discussion

In this study, we use next-generation RNA sequencing in PsA blood and skin fibroblasts to characterize the transcriptomic landscape of the disease. We define molecular signatures associated with disease activity and early response to treatment, using longitudinal transcriptomic and immunophenotypic analysis. Additionally, our data are suggestive of a dynamic cross-talk between blood immune cells and skin fibroblasts, highlighting novel pathways of pathophysiologic relevance.

We identified an ‘*activity signature*’ in PsA blood pertaining to the regulation and response of the immune system. The TNFα signaling pathway was enriched in PsA patients compared to HI, confirming the role of this cytokine in disease pathophysiology and the importance of its therapeutic targeting (31, 32). Furthermore, an IFN response signature related to both type-I and type-II interferon was also identified in PsA blood, corroborating the findings of a former microarray study (33). IFN-α is a crucial effector molecule that bridges innate with adaptive immunity and its overexpression has been identified as a key driver in many systemic autoimmune diseases (34). Of note, excessive type-I IFN activity was recently revealed in the blood of patients with general pustular PsO (29) suggesting a systemic activation of the IFN system in psoriatic disease. Intriguingly, we did not observe a signature directly related to the IL-17/IL-23 axis, despite its key role in PsA pathogenesis (35, 36). Nevertheless, we observed an overexpression of transcripts related to TGFβ signaling, which could indirectly lead to activation of this pathogenic cytokine axis, given its crucial role in promoting Th17 cell differentiation (37).

Phenotypic analysis of blood also revealed aberrancies in the immune cell profile of subjects with active PsA. Specifically, we observed significantly increased NCM in affected compared to HI at baseline, and persistent increase of these cells in patients who failed to respond to treatment. NCM display a highly pro-inflammatory phenotype (38) and enhanced antigen presenting capacity that may sustain effector immune responses in treatment-resistant PsA. These cells have vascular-patrolling features (39) and may contribute to PsA-related vascular comorbidities such as atherosclerosis and cardiovascular disease (40). A recent study using high-throughput techniques demonstrated enrichment of the CD14^+^ monocytic population in PsA joints, corroborating the role of these cells as drivers of inflammation in PsA through the secretion of pro-inflammatory mediators (9). Our immunophenotypic analysis also revealed decreased frequency of circulating pDCs and cytotoxic T cells, consistent with former observations suggesting recirculation of these cells between blood and PsA target tissues (41, 42).

Our data also suggested a contribution of aberrant lipid-related metabolism to the PsA ‘activity signature’. PsA patients show an increased prevalence of cardiovascular risk factors (hypertension, obesity, insulin resistance) (5, 43) and an aberrant blood lipid profile that favors immune-system modulating mediators (COX-2, PGE1, LTB4) (44). In line with these observations, we identified a deregulated lipidomic signature in peripheral blood of PsA patients characterized by upregulation of transcripts related to cholesterol and fatty acid metabolism. We also identified enhanced signaling mediated by mTOR, a primary regulator of metabolic reprogramming in T cells and a key driver in T-cell mediated autoimmune diseases (45).

PsA is uniquely characterized by synchronous bone erosions and pathological new bone formation (46). Elevated extracellular matrix (ECM) protein fragments in PsA serum reflect the deregulated bone and cartilage turnover and bear the potential to function as diagnostic biomarkers (46–48). In agreement with these observations, our study also provided evidence of aberrant collagen metabolism in PsA blood during the active phase of the disease. By WGCNA analysis in PsA patients and HI, we identified one gene module (orange) that was mainly enriched in ECM structure and organization processes. Interestingly, this module exhibited a negative correlation with PsA, implying the possible efflux of circulating mesenchymal-like cells from the blood to affected tissues. This finding is in accordance with a recent study that highlighted the role of blood circulating mesenchymal cells (PRIME cells) in mediating a RA flare (11). Importantly, we identified that this ECM-related gene signature, comprising 67 genes, was highly specific for PsA, after comparisons with the blood transcriptome profile of subjects who have RA, PsO and SLE.

Skin is an important target tissue in PsA and therefore, we sought to explore the contribution of dermal fibroblasts -the main regulators of ECM homeostasis- in disease pathogenesis. Our transcriptomic analysis revealed that PsA fibroblasts exhibited greater proliferation and increased mitochondrial respiration compared to fibroblasts from HI, which could underpin their activated phenotype (49, 50). Notably, PsA fibroblasts also displayed decreased expression of transcripts involved in TNFα signaling and IFN response, possibly shaping a counteracting immunosuppressive adaptation to the inflammatory microenvironment of the psoriatic skin. This finding comes in contrast to a recent proteomic study by Gegotek et al. (51), who demonstrated increased production of pro-inflammatory molecules such as TNFα and NFkB from psoriatic fibroblasts. Nevertheless, this difference should be considered with caution, given the different types of molecules analyzed in the two studies. We also identified, for the first time, an aberrant communication network between skin fibroblasts and blood cells in PsA. This network included, among others, enhanced WNT-mediated signaling from blood to skin as well as enhanced autocrine NOTCH signaling in PsA fibroblast. Both of these signaling pathways have a key role in promoting myofibroblast differentiation in fibrotic diseases (52, 53), and their identification has implications for PsA pathogenesis.

An interesting question raised by this study is whether there are transcriptomic or immunophenotypic signatures in blood that could predict response to treatment in PsA. Blood is an easily accessible tissue that could serve as a valuable source of prognostic biomarkers. Although serum levels of acute-phase reactants and complement components have been described to associate with the ACR50 response criteria in PsA (54), there are no molecular biomarkers implemented in the routine clinical care (55). Responders and non-responders in our study did not present significant differences related to their baseline and 1-month clinical characteristics. Both groups also did not show significant differences in their blood immune profile at the different time points. On the other hand, the longitudinal immunophenotypic analysis revealed increased frequency of NCM at the end of the follow-up (6-month) compared to the 1-month time point within the group of non-responders. Additionally, the longitudinal transcriptomic analysis revealed that implementation of treatment had significantly different effect on the biological pathways in the two patient groups. Specifically, we showed that pathways related to TGFβ signaling and angiogenesis were upregulated at 6-months compared to baseline in non-responders. On the other hand, responders demonstrated downregulation of biological processes related to inflammation, such as TNFα signaling, IFNα and IFNγ response. These results support the notion that aberrant TGFβ signaling and the Ag-presenting capacity of NCM may sustain Th17 effector cells in treatment-resistant PsA. This model is consistent with the observations that TGFβ is an important activator of the IL-23/IL-17 pathway (56) and that NCM can trigger the induction of the pro-inflammatory Th17 cells (57). Finally, the blood transcriptomic profile of responders diverged from that of non-responders at the 1-month time point, providing thus an early transcriptomic signature of prognostic potential. This signature consisted of genes related to platelet activation and MST1-mediated Hippo signaling. These findings are of important pathophysiological relevance, given the central role of Hippo pathway in regulating immune responses such as NFκB signaling (58) and IFN production (59) as well as the emerging role of platelets as pro-inflammatory mediators in autoimmune diseases (60).

Our study has certain limitations. First, the sample size of our study is small, pending validation of our findings in larger cohorts. The molecular signature associated with response to treatment should also be confirmed in an independent longitudinal cohort. Secondly, both PsA and RA patients displayed significant heterogeneity regarding their background treatment at the time of blood collection. Although our initial approach was to recruit homogeneous patient groups, difficulties related to the COVID-19 pandemic made this endeavor challenging. We recognize that our cohort heterogeneity may represent a confounding factor limiting the replicability of our results. Yet, as all participants had highly active disease at enrollment, we believe that the molecular signatures of ‘activity’ and ‘response to treatment’ identified are independent of the background therapy. Future studies focusing on specific patient groups could provide more insight into this issue. Of note, subgroup analyses comparing naive patients, patients on DMARDs and patients on biologics at baseline did not reveal significant differences among the groups (data not shown). Finally, our study focused on patients with chronic, established PsA, and thus, our findings cannot be generalized to patients with early disease.

In summary, by the use of combined transcriptomic and immunophenotypic analysis, we define an ‘*activity signature*’ in PsA blood characterized by TNF- and IFN-driven inflammation, lipid-related metabolic aberrancies and expansion of pro-inflammatory non-classical monocytes. Our data also suggest the presence of a “*PsA-specific gene set*” enriched in ECM metabolism, supported by an enhanced communication network between blood immune cells and skin resident mesenchymal cells. Finally, we also provide evidence of persistent TGFβ signaling and angiogenesis in treatment-resistant PsA, and propose a gene expression signature related to platelet activation and Hippo signaling as potential biomarker of early response to treatment. These findings highlight the significant molecular heterogeneity of PsA and may open new avenues, including a novel molecular characterization of patients, better risk stratification strategies, and more efficient tailoring of treatment.

## Data availability statement

The datasets presented in this study can be found in online repositories. The names of the repository/repositories and accession number(s) can be found below: https://ega-archive.org/, EGAS00001006288.

## Ethics statement

This study was reviewed and approved by Attikon University Hospital Research Ethic Committee (Athens, Greece, protocol 103/6-3-2014). The patients/participants provided their written informed consent to participate in this study.

## Author contributions

AG and MG designed and performed the experiments, analyzed data, generated figures and wrote the manuscript. NM participated in the preparation of the libraries. GS and AF analyzed data and generated figures. AG, SF and PK performed clinical evaluation of patients and provided human specimens. PV participated in the design, the interpretation of data and the editing of the manuscript. DB supervised the study, analysis of data and the writing of the manuscript. All authors contributed to the article and approved the submitted version.

## Funding

This work was supported by a research grant from the European Research Council (ERC) under the European Union’s Horizon 2020 research and innovation program (grant agreement No 742390). Computational time granted from the National Infrastructures for Research and Technology S.A. (GRNET S.A.) in the National HPC facility - ARIS - under project ID pr007035_thin-SLE_BIO.

## Acknowledgments

We thank Anastasia Apostolidou from the Flow Cytometry Facility (BRFAA) for cell sorting, Dr. Ioannis Vatsellas from the Greek Genome Center (BRFAA) for the next-generation sequencing service and Dr. Eirini Kapniari (Attikon University Hospital) for assistance with skin biopsies. We also thank the patients and their referring physicians and nurses at the Attikon Rheumatology Unit.

## Conflict of interest

The authors declare that the research was conducted in the absence of any commercial or financial relationships that could be construed as a potential conflict of interest.

## Publisher’s note

All claims expressed in this article are solely those of the authors and do not necessarily represent those of their affiliated organizations, or those of the publisher, the editors and the reviewers. Any product that may be evaluated in this article, or claim that may be made by its manufacturer, is not guaranteed or endorsed by the publisher.

## Supplementary material

Supplementary Figure 1 Deconvolution, gating strategy and immunophenotyping in RA blood samples. **(A)** Violin diagram illustrating the fraction of selected immune cell subsets in the peripheral blood of PsA patients (n=23) and HI (n=7), as inferred by CIBERSORTx. **(B)** Representative gating strategy for the identification of myeloid and lymphoid cell subsets. Classical monocytes were characterized as HLA-DR+CD14+CD16-, intermediate monocytes as HLA-DR+CD14+CD16+, non-classical monocytes as HLA-DR+CD14-CD16+ and pDCs as HLA-DR+CD14-CD16-CD123+. T helper cells and T cytotoxic cells were identified as CD4+CD8- and CD4-CD8+, respectively. CD4+ cells were further gated for CD25+ T regulatory cells. B cells were identified as CD19+ cells. **(C)** Frequencies of monocyte subsets, pDCs, B cells, Tregs, CD4+ and CD8+ T cells in peripheral blood of PsA (n= 26-30), RA (n=8-10) and HI (n= 8-10). Results are demonstrated as mean with SD. Statistical significance was obtained by unpaired Student’s t-test and Mann-Whitney test. (*p ≤ 0.05, **p ≤ 0.01, ***p ≤ 0.001). RA, Rheumatoid arthritis; pDCs, Plasmacytoid dendritic cells; Tregs, T regulatory cells; PsA, Psoriatic arthritis; HI, Healthy individuals.

Supplementary Figure 2 Blood transcriptome analysis in RA samples. **(A)** PCA of blood gene expression profiles from PsA, RA. The two first principal components (PC1, PC2) are plotted. **(B)** Volcano plot (left) and heatmap (right) of DEGs between PsA and RA. The up- and down-regulated genes are denoted by red and blue points, respectively. Gray points indicate genes with no significant difference in expression between PsA and RA. RA, Rheumatoid arthritis; PCA, Principal component analysis; PsA, Psoriatic arthritis; HI, Healthy individuals; DEGs, Differentially expressed genes.

Supplementary Figure 3 Comparison of PsA and SLE blood gene expression profile. **(A)** Venn diagram showing the overlap among significant DEGs of ‘SLE vs. HI’, ‘PsA vs. HI’, and ‘PsA-specific gene set’. 57 out of the 67 “PsA-specific gene set” are uniquely represented in the ‘PsA vs. HI’ comparison. PsA, Psoriatic arthritis, SLE, Systemic lupus erythematosus; DEGs, Differentially expressed genes; HI, Healthy individuals

Supplementary Figure 4 Transcriptomic analysis of skin fibroblasts. **(A)** Heatmap (left) and volcano plot (right) of DEGs between PsA (n=3) and HI (n=3). The up- and down-regulated genes are denoted by red and blue points, respectively. Gray points indicate genes with no significant difference in expression between PsA and HI. **(B)** Dot plot of GSEA analysis (Hallmark v7.5) representing biological pathways associated with PsA fibroblasts. The figure shows the significantly positively and negatively enriched terms. **(C)** Bar plot indicating the overall number and strength of communication interactions between skin fibroblasts and blood cells in PsA and HI. DEGs, Differentially expressed genes; PsA, Psoriatic arthritis; HI, Healthy individuals; GSEA, Gene set enrichment analysis; FC, Fold change.

Supplementary Figure 5 Transcriptomic analysis in Responders and Non-Responders during the 6-month treatment period. **(A)** Volcano plots of DEGs between baseline (0-) and 6-month in R (left) and NR (right). **(B)** Volcano plots of DEGs (|FC|>1.5, P value<0.05) between R and NR at baseline (left), 1- (middle) and 6- (right) after treatment initiation. The up- and down-regulated genes are denoted by red and blue points, respectively. **(C)** Dot plot representing pathway analysis of DEGs from PsA ‘R vs. NR’ at baseline (left) and at 6-month time point (right) based on g:Profiler. DEGs, Differentially expressed genes; R, Responders; NR, Non-responders; PsA, Psoriatic arthritis.
