# Supplementary figures and images for: Combined – whole blood and skin fibroblasts- transcriptomic analysis in Psoriatic Arthritis reveals molecular signatures of activity, resistance and early response to treatment
Source: Front Immunol. 2022 Sep 8;13:964274. doi: 10.3389/fimmu.2022.964274 (PMC9493103; doi:10.3389/fimmu.2022.964274)

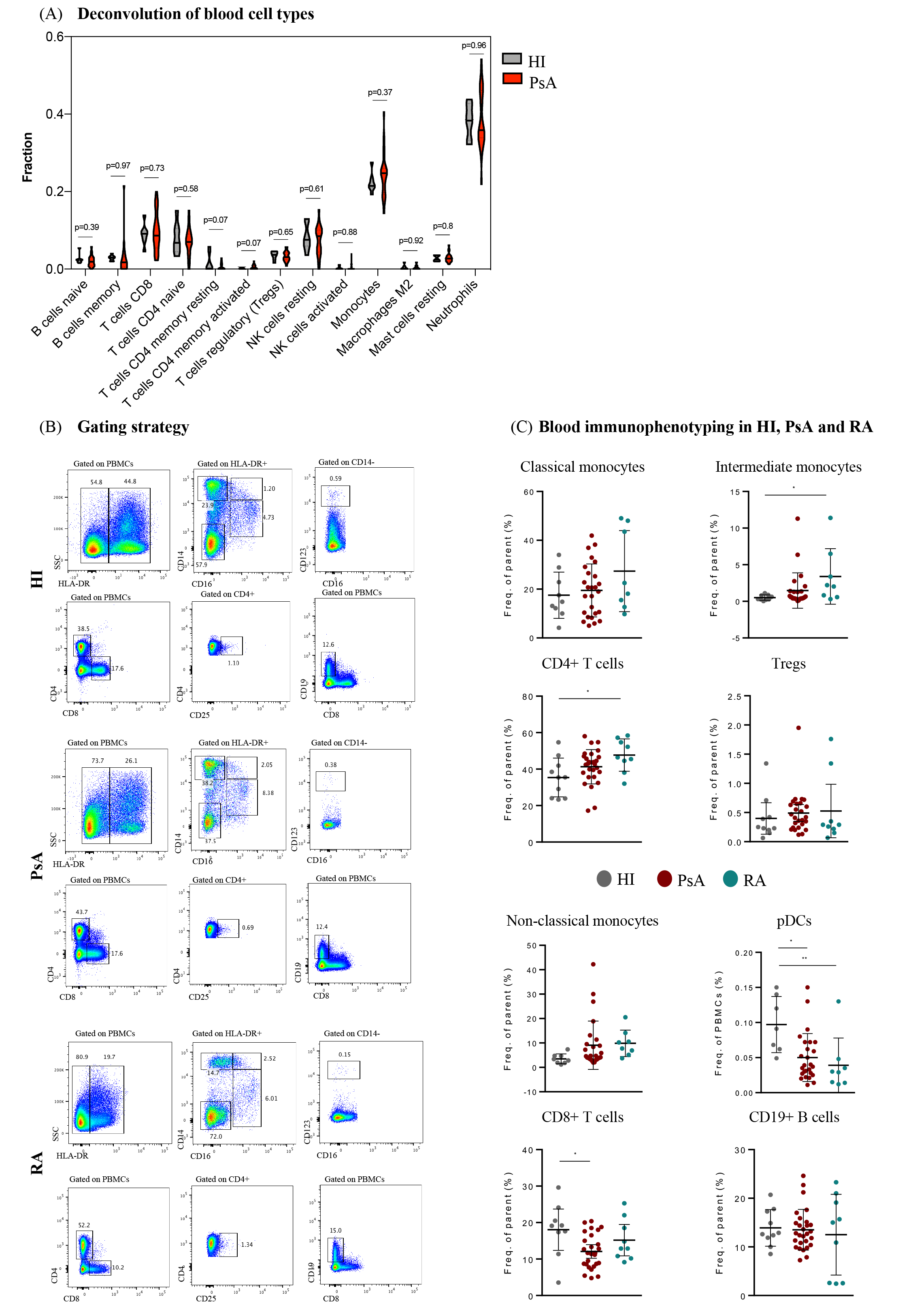

Supplement: Supplementary Figure 1 — Deconvolution, gating strategy and immunophenotyping in RA blood samples. (A) Violin diagram illustrating the fraction of selected immune cell subsets in the peripheral blood of PsA patients (n=23) and HI (n=7), as inferred by CIBERSORTx. (B) Representative gating strategy for the identification of myeloid and lymphoid cell subsets. Classical monocytes were characterized as HLA-DR+CD14+CD16-, intermediate monocytes as HLA-DR+CD14+CD16+, non-classical monocytes as HLA-DR+CD14-CD16+ and pDCs as HLA-DR+CD14-CD16-CD123+. T helper cells and T cytotoxic cells were identified as CD4+CD8- and CD4-CD8+, respectively. CD4+ cells were further gated for CD25+ T regulatory cells. B cells were identified as CD19+ cells. (C) Frequencies of monocyte subsets, pDCs, B cells, Tregs, CD4+ and CD8+ T cells in peripheral blood of PsA (n= 26-30), RA (n=8-10) and HI (n= 8-10). Results are demonstrated as mean with SD. Statistical significance was obtained by unpaired Student’s t-test and Mann-Whitney test. (*p ≤ 0.05, **p ≤ 0.01, ***p ≤ 0.001). RA, Rheumatoid arthritis; pDCs, Plasmacytoid dendritic cells; Tregs, T regulatory cells; PsA, Psoriatic arthritis; HI, Healthy individuals. [file Image_1.tif]

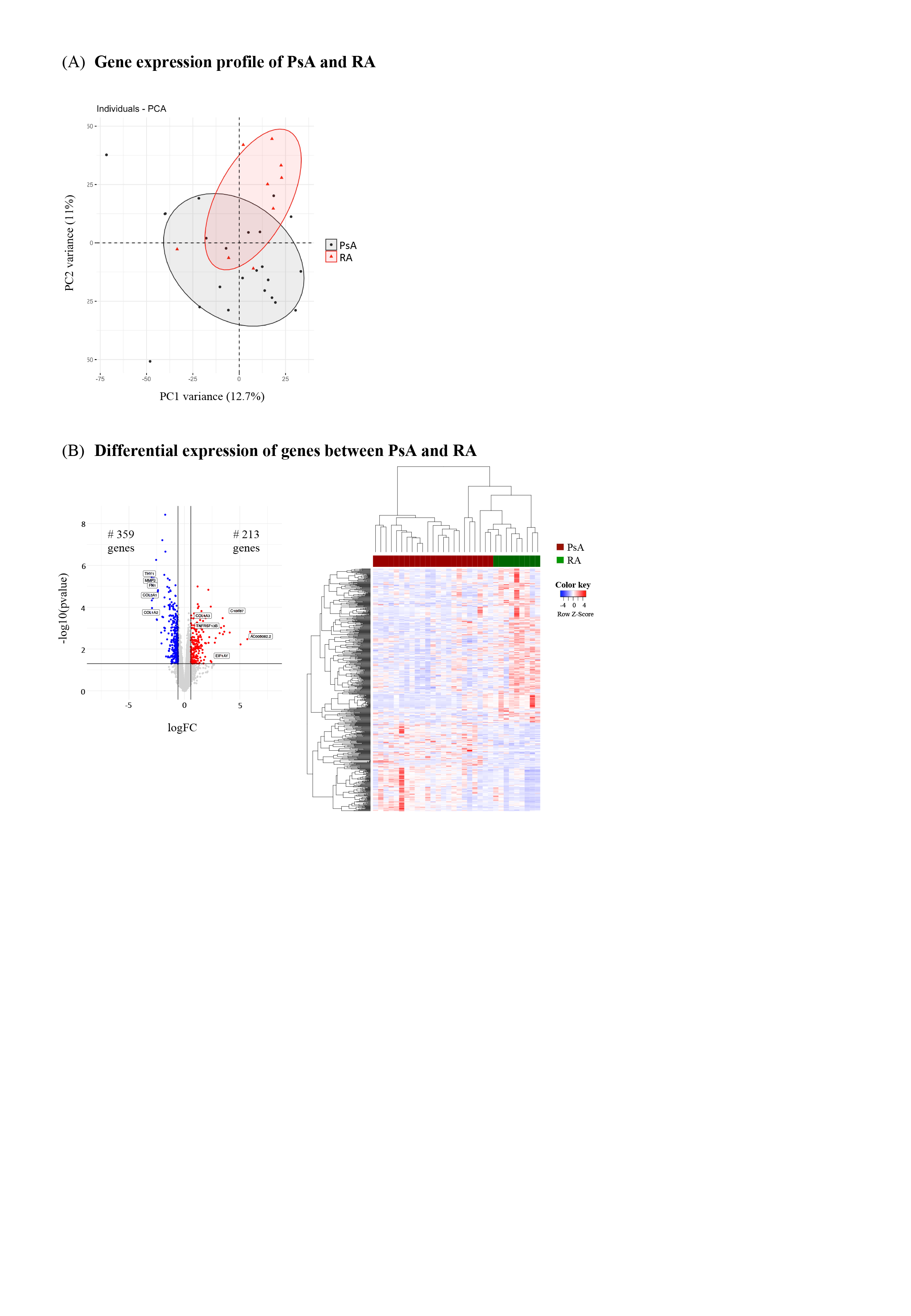

Supplement: Supplementary Figure 2 — Blood transcriptome analysis in RA samples. (A) PCA of blood gene expression profiles from PsA, RA. The two first principal components (PC1, PC2) are plotted. (B) Volcano plot (left) and heatmap (right) of DEGs between PsA and RA. The up- and down-regulated genes are denoted by red and blue points, respectively. Gray points indicate genes with no significant difference in expression between PsA and RA. RA, Rheumatoid arthritis; PCA, Principal component analysis; PsA, Psoriatic arthritis; HI, Healthy individuals; DEGs, Differentially expressed genes. [file Image_2.tif]

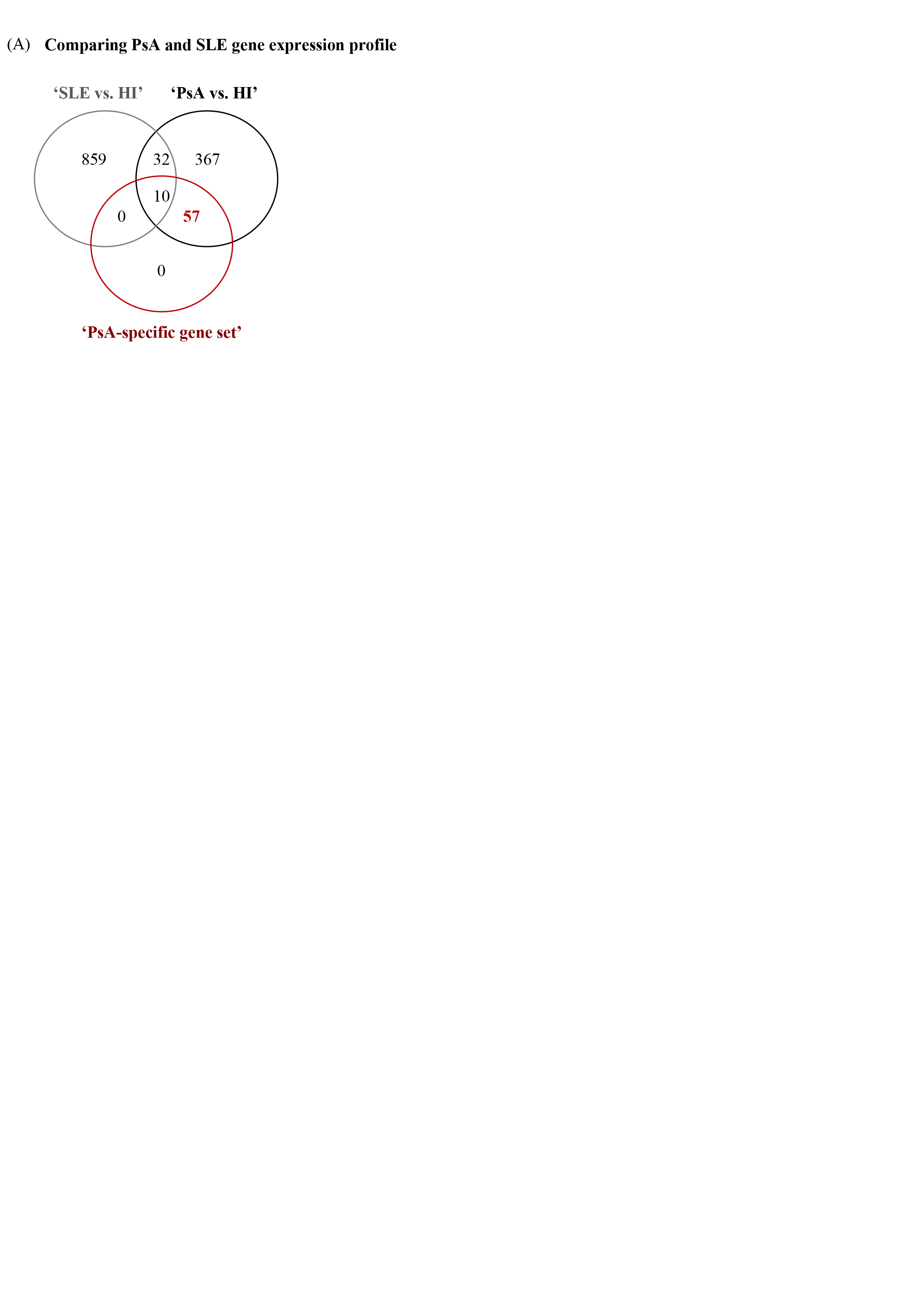

Supplement: Supplementary Figure 3 — Comparison of PsA and SLE blood gene expression profile. (A) Venn diagram showing the overlap among significant DEGs of ‘SLE vs. HI’, ‘PsA vs. HI’, and ‘PsA-specific gene set’. 57 out of the 67 “PsA-specific gene set” are uniquely represented in the ‘PsA vs. HI’ comparison. PsA, Psoriatic arthritis, SLE, Systemic lupus erythematosus; DEGs, Differentially expressed genes; HI, Healthy individuals [file Image_3.tif]

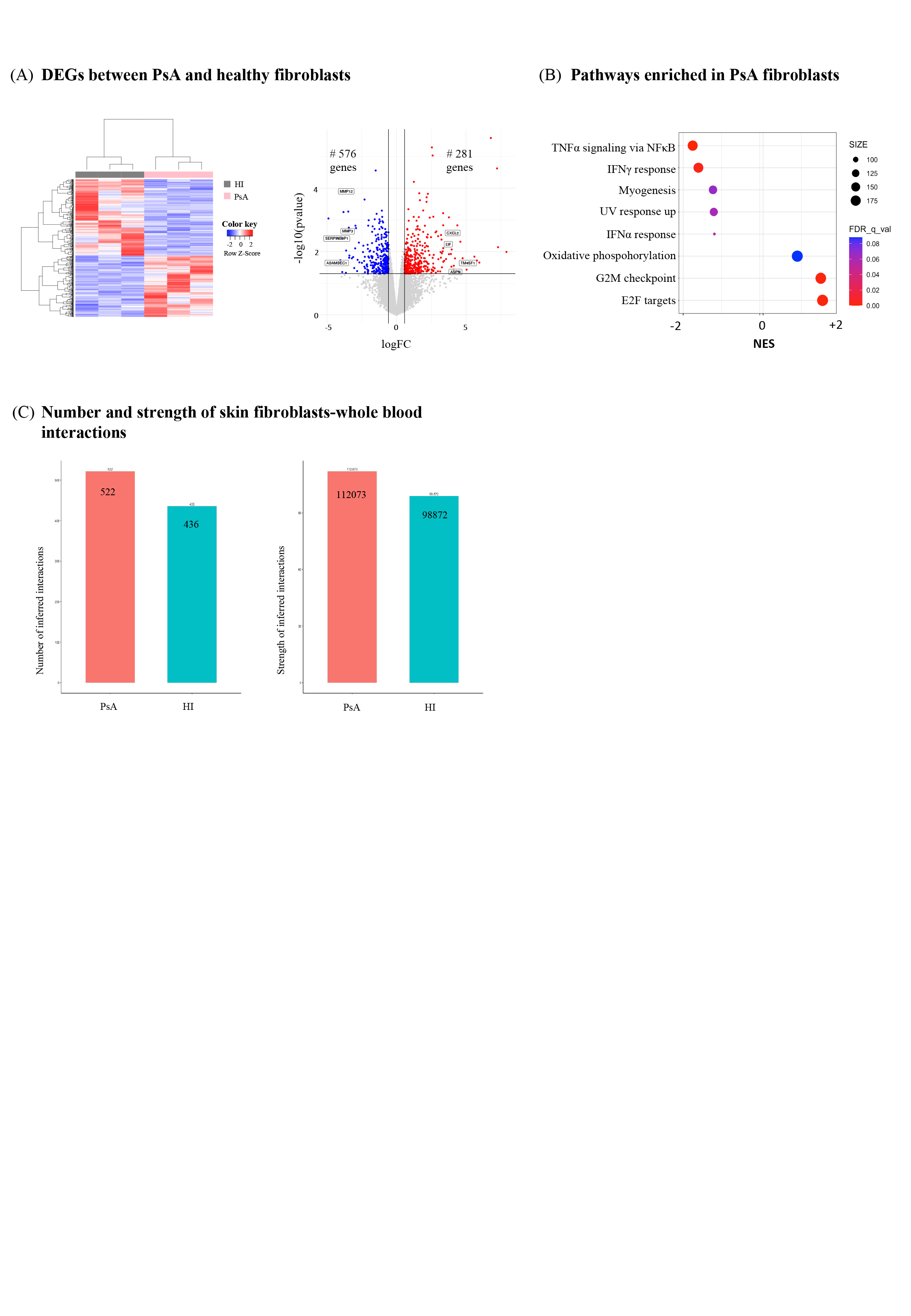

Supplement: Supplementary Figure 4 — Transcriptomic analysis of skin fibroblasts. (A) Heatmap (left) and volcano plot (right) of DEGs between PsA (n=3) and HI (n=3). The up- and down-regulated genes are denoted by red and blue points, respectively. Gray points indicate genes with no significant difference in expression between PsA and HI. (B) Dot plot of GSEA analysis (Hallmark v7.5) representing biological pathways associated with PsA fibroblasts. The figure shows the significantly positively and negatively enriched terms. (C) Bar plot indicating the overall number and strength of communication interactions between skin fibroblasts and blood cells in PsA and HI. DEGs, Differentially expressed genes; PsA, Psoriatic arthritis; HI, Healthy individuals; GSEA, Gene set enrichment analysis; FC, Fold change. [file Image_4.tif]

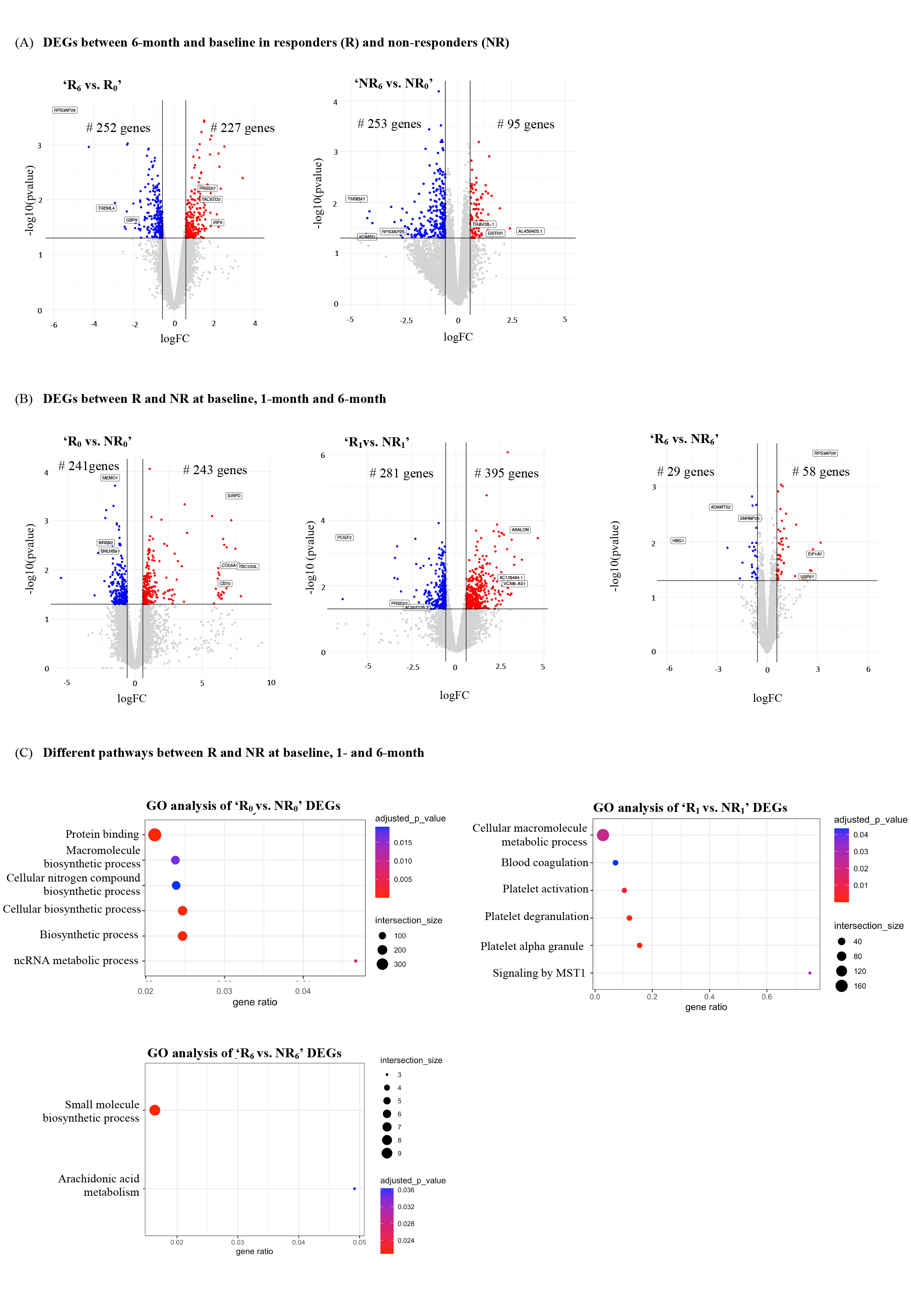

Supplement: Supplementary Figure 5 — Transcriptomic analysis in Responders and Non-Responders during the 6-month treatment period. (A) Volcano plots of DEGs between baseline (0-) and 6-month in R (left) and NR (right). (B) Volcano plots of DEGs (|FC|>1.5, P value<0.05) between R and NR at baseline (left), 1- (middle) and 6- (right) after treatment initiation. The up- and down-regulated genes are denoted by red and blue points, respectively. (C) Dot plot representing pathway analysis of DEGs from PsA ‘R vs. NR’ at baseline (left) and at 6-month time point (right) based on g:Profiler. DEGs, Differentially expressed genes; R, Responders; NR, Non-responders; PsA, Psoriatic arthritis. [file Image_5.tif]
